# Preparation and Performance of Ternesite–Ye’elimite Cement

**DOI:** 10.3390/ma15124369

**Published:** 2022-06-20

**Authors:** Yan Shen, Xi Chen, Jiang Li, Peifang Wang, Jueshi Qian

**Affiliations:** 1 College of Building and Materials, Chongqing College of Electronic Engineering, Chongqing 401331, China; cqlcm@126.com; 2 College of Civil Science and Engineering, Yangzhou University, Yangzhou 225127, China; 18252775161@163.com (X.C.); wpf_0149@163.com (P.W.); 3 College of Material Science and Engineering, Chongqing University, Chongqing 400045, China; qianjueshi@126.com

**Keywords:** ternesite–ye’elimite cement, doping, clinkering, hydration, compressive strength

## Abstract

Ternesite–ye’elimite (TCSA) cement is a new type of environmentally advantageous binder prepared by introducing ternesite, a reactive phase, into belite calcium sulfoaluminate cement clinker. This paper reports the laboratory production of TCSA cement by the addition of minor elements to achieve the coexistence of ternesite and ye’elimite. The influence of dopants on the mineralogical composition of clinkers and the clinkering conditions for the preparation of TCSA cement clinkers were investigated by X-ray powder diffraction and scanning electron microscopy. The mechanical properties and hydration products of the cement pastes were also studied. The results indicated that the addition of CaF_2_, P_2_O_5_ and Na_2_O can promote the coexistence of ternesite and ye’elimite, and that Na_2_O is the most effective candidate. TCSA cement clinkers could be successfully prepared at 1150 °C for 30 min by doping 0.3% Na_2_O. The TCSA cement clinkers exhibited shorter setting times than the BCSA cement clinkers. The later strength of TCSA cement showed a significant increase compared with BCSA cement. The effect of Na_2_O was different on the strength development for TCSA and BCSA cement. The dissolution of ternesite could promote the formation of ettringite. The reactivity of belite was higher in TCSA cement due to the formation of strätlingite.

## 1. Introduction

Portland cement (PC) is extensively used in construction engineering all over the world. However, the cement industry has the disadvantage of consuming a large amount of energy, and it generates 5~10% of anthropogenic CO_2_ emissions [1,2,3]. Therefore, the development of new types of environmentally advantageous cements is highly expected to reduce the environmental load. Calcium sulfoaluminate (CSA) cement was proposed as a sustainable alternative to PC owing to reductions in CO_2_ emission and energy consumption [4,5]. CSA cement clinker is produced by calcining limestone, bauxite and gypsum at 1250–1350 °C, which is lower than the temperature for PC production. Therefore, CSA cement has significant environmental benefits. Typically, CSA cement clinkers consist of more than 50 wt.% of ye’elimite ($C_{4}A_{3}\bar{S}$), belite (C_2_S) and other compounds, including gehlenite (C_2_AS), anhydrite ($C\bar{S}$), ferrite (C_4_AF), periclase (M), mayenite (C_12_A_7_) and perovskite (CT) [6,7]. CSA cements have exhibited rapid setting, high early-age strength, low permeability and shrinkage compensation [8]. The applications of CSA cements are mainly in pre-cast concrete, small-scale repair products, and glass-fiber-reinforced composites in China. However, the scarcity of aluminum material, its high cost, and strength deterioration limit the application of this kind of cement on a large scale.

Recently, a subclass of belite-rich CSA cement, known as belite calcium sulfoaluminate (BCSA) cement, has received increasing attention [9,10]. This kind of cement contains the dominant mineral belite and the secondary phase ye’elimite. Hence, the production of BCSA cement allows low-grade bauxite and aluminum bearing solid wastes to be used to decrease the demand for bauxite, resulting in lower production costs. Due to the high reactivity of $C_{4}A_{3}\bar{S}$, BCSA cement has high early strength. However, the compressive strength of BCSA cement increases slowly at later ages because of the slow hydration reactivity of belite [11]. During the clinkering process of CSA-based cements, belite can react with anhydrite to form a transitory phase, ternesite $C_{5}S_{2}\bar{S}$, at temperatures higher than 900 °C [12]. Ternesite was previously identified as a slowly hydrating phase [13,14,15,16]. Recently, ternesite has gained increasing interest because of its hydraulic reactivity when present in CSA cements [17]. Moreover, ternesite even showed higher reactivity than belite [17]. Therefore, the creation of a new type of cement clinker based on ternesite and ye’elimite is proposed by introducing ternesite into BCSA cement clinker in this paper.

The formation of ternesite was mainly dependent on the clinkering temperature. Hou et al. showed that ternesite was stable within a temperature range of 1100 to 1200 °C [6]. A temperature higher than 1200 °C was able to decompose ternesite to belite and anhydrite. However, the CSA cement clinkers were manufactured in the temperature range of 1250 to 1350 °C in order to ensure the full formation of ye’elimite. Therefore, the formation/decomposition temperature of ternesite and ye’elimite was incompatible. Some approaches have been reported to realize the coexistence of ternesite and ye’elimite. Bullerjahn et al. used a two-stage clinkering process, firstly clinkering at 1250 °C for 1 h and then clinkering at 1100 °C for 1 h, in order to enhance the formation of ternesite [17]. Similarly, Shen et al. studied the laboratory production of belite-sulfoaluminate-ternesite cements by adopting two successive sintering steps [18]. A first clinkering for 30 min at 1270 °C and a second clinkering for 1 h at 1100–1200 °C were carried out to stabilize ternesite as a clinker component. The formation of ternesite was reported to improve the mechanical strength at later ages. Hanein et al. produced ternesite-rich calcium sulfoaluminate clinkers in a single burning process at a temperature above 1200 °C by controlling the partial pressures of SO_2_ and O_2_ as well as the temperature [19]. Ji et al. determined the best sintering temperature and sintering time of belite-ye’elimite-ternesite cement clinker by setting a temperature point every 30 °C (1150–1300 °C) and setting every hour (1–6 h) [20]. This clinker could be obtained by sintering at 1210 °C for 2 h. Li et al. prepared ternesite–ye’elimite clinker from steel slag at 1200 °C [21]. The iron phase from steel slag can promote the formation of the liquid phase, and can thereby result in the coexistence of ternesite and ye’elimite. Mineralizers, such as phosphates or fluorides, were usually used during the clinkering process to promote mineral formation. When ternesite was stabilised at temperatures higher than 1200 °C, the use of mineralizers was necessary [22]. Skalamprinos et al. investigated the influence of dopants on the synthesis and hydration of ternesite [23]. It was found that the most effective promotion of ternesite formation was obtained by the addition of MgO in the precedence of 0.2% K_2_O and 0.1% Na_2_O. Shen et al. found that among the five dopants (CaF_2_, Na_2_O, Fe_2_O_3_, MgO, P_2_O_5_), CaF_2_ was most effective in the promotion of ternesite formation [24]. Therefore, adding minor elements is expected to promote the coexistence of ternesite and ye’elimite.

The objective of this study is to produce ternesite–ye’elimite (TCSA) cement clinkers in a single stage process. Considering the discrepancy of the formation/decomposition temperature of ternesite and ye’elimite, this paper employs minor elements to expand the coexisting temperature range of the two clinker phases. The influence of dopants on the formation of ternesite and ye’elimite was investigated for the synthesis of TCSA cement clinkers in a one-step process. Moreover, the parameters affecting the formation of ternesite—such as the gypsum content in raw mixes, sintering temperature and time—were also studied. This study is intended to further aid the understanding of the mechanical properties and the hydration process. The final TCSA cements are expected to develop better performance than the BCSA cements. This study will provide a new thought for the synthesis of TCSA clinkers.

## 2. Experiment

### 2.1. Raw Materials

For the production of the TCSA cement clinkers, the raw materials used were limestone, bauxite, fly ash and natural gypsum. These materials were obtained from the market in Jiangsu, China. The chemical compositions of the materials, as measured by X-ray fluorescence spectrometry (XRF), are listed in Table 1. The dopants used were analytical grade: CaF_2_, Ca_3_(PO_4_)_2_, Fe_2_O_3_, MgO and Na_2_CO_3_. All of the materials were dried and ground in a laboratory mill until the 100% passing of the 75-μm sieve.

### 2.2. Synthesis of the TCSA Cement Clinkers

**Figure 1:**
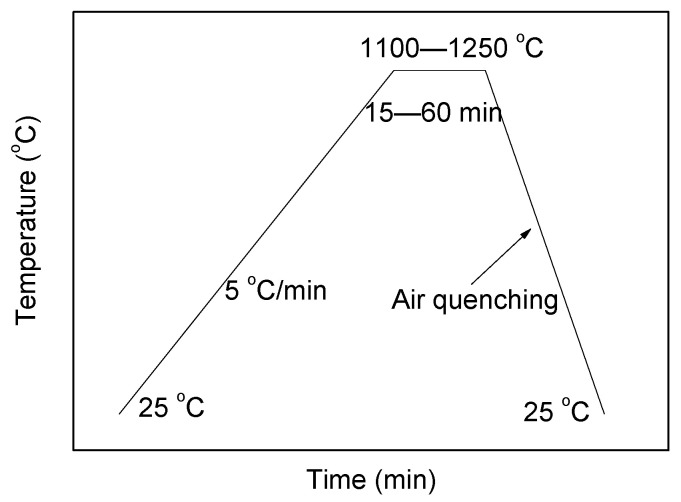
Sintering profiles for the preparation of the TCSA cement clinkers.

Three TCSA cement clinkers were synthesized in this study. The targeted compositions of the TCSA cement clinkers are demonstrated in Table 2. The total amount of ternesite, belite and anhydrite was kept constant (50%) in clinkers A, B and C, while the content of ye’elimite was 40%. Based on the reaction degree of the anhydrite with belite during clinkering process, the contents of anhydrite in clinkers A, B and C were insufficient, moderate (2%) and excessive (8%), respectively. The mix proportions of limestone, bauxite, gypsum and fly ash are given in Table 3. For clinkers B and C, additional analytical-grade Al_2_O_3_ was added to the raw mixes because the provision of aluminum from bauxite was not sufficient. The dopant additions, with the proportions of the total amount of raw materials, are shown in Table 4. All of the materials were homogenized using a mixer for 1 h, and then the mixtures were made into disk models (φ 50 mm × 8 mm). After being dried in an oven (50 °C for 24 h), the samples were placed into corundum crucibles and heated in a furnace to temperatures between 1100 and 1250 °C for 15–60 min (Figure 1). Finally, the clinkers were quenched with forced air. The clinkers were ground using a ball mill to 100% pass the 75-μm sieve.

### 2.3. Testing Methods

Pastes were produced using a water/cement ratio of 0.5. The setting time was determined using a standard Vicat apparatus in accordance with Chinese standard GB/T 1346–2001. The compressive strengths were tested on 20-mm cubic samples. The cement pastes were cured at a temperature of 20 °C and a humidity of 95%. After 1 day of curing, they were demoulded and continuously cured in water at 20 °C. The compressive strength tests were conducted at the hydration ages of 1, 3, 7 and 28 days on six samples. The strength value was the average of six samples.

The hydrated samples were crushed and subsequently submerged in ethanol for 24 h to prevent further hydration. Finally, the samples were dried at 40 °C and milled to pass a 75-μm sieve. XRD (Bruker D8 Advance diffractometer with Cu Kα radiation, Bilerica, MA, USA) was used to analyse the mineralogy of the synthesized clinkers and hydrated pastes. The instrument was operated at 40 kV and 40 mA with a step size of 0.02°. The quantitative information of the clinkers was obtained through the Rietveld method with TOPAS 4.2 software (Version 3, Bilerica, MA, USA). The crystal structures for the Rietveld analysis were reported as cubic-$C_{4}A_{3}\bar{S}$(PDF# 071-0969), ortho-$C_{4}A_{3}\bar{S}$ (PDF# 085-2210), β-C_2_S (PDF# 086-0398), C_2_F (PDF# 038-0408), C_4_AF (PDF# 071-0667), $C\bar{S}$ (PDF# 074-1639) and $C_{5}S_{2}\bar{S}$ (PDF# 070-1847). The morphological features of the specimens were characterizes by means of a GeminiSEM 300 Scanning Electron Microscope (SEM, Hitachi, Tokyo, Japan). The polished cross sections were coated with gold in order to obtain a conductive surface for observations. Thermogravimetic analysis (TG-DSC) was carried out using a TG/DSC1/1600LF thermal analyzer (Mettler Toledo, Zurich, Switzerland). The samples were heated from 30 °C to 1000 °C with a heating rate of 10 °C/min under a nitrogen atmosphere (flow rate 50 mL/min).

## 3. Results and Discussion

### 3.1. Synthesis of the TCSA Cement Clinkers

#### 3.1.1. Influence of Dopants on the Coexistence of Ternesite and Ye’elimite

**Figure 2:**
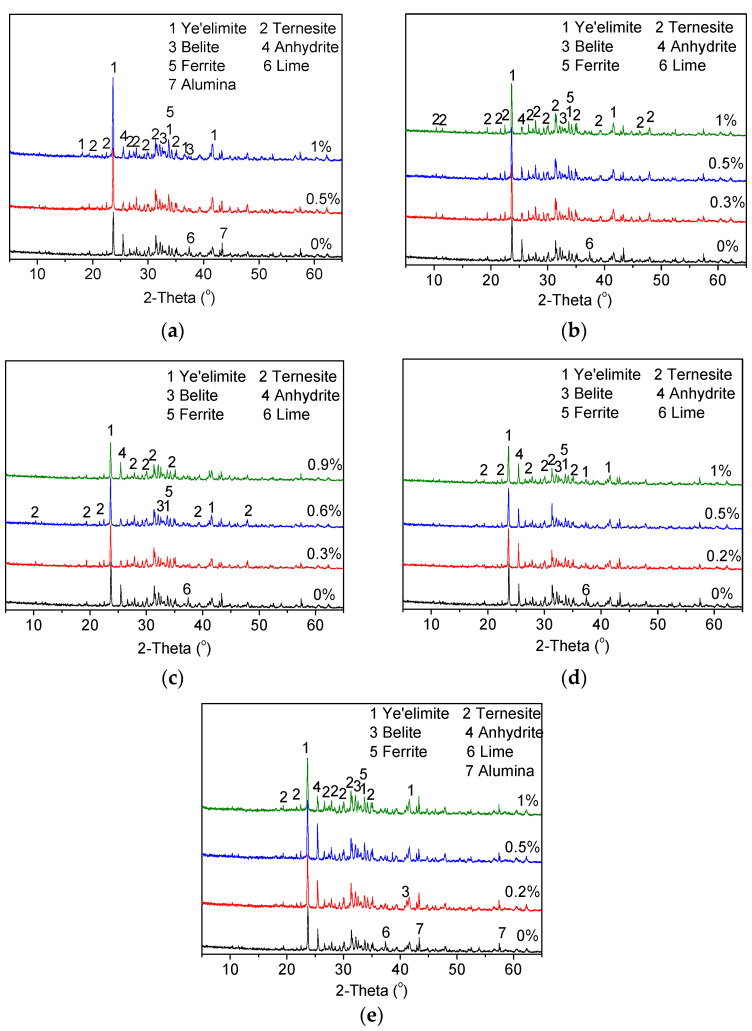
XRD patterns of doped cement clinker A. (**a**) CaF_2_; (**b**) P_2_O_5_; (**c**) Na_2_O; (**d**) MgO; (**e**) Fe_2_O_3_.

The addition of the dopants had a significant influence on the formation of ternesite as a single phase [23,24]. Therefore, some minor elements may promote the coexistence of ternesite and ye’elimite. The influence of dopants on the phase composition of clinker A sintered at 1150 °C for 30 min are presented in Figure 2. The main minerals of clinker A were ye’elimite, ternesite, belite, anhydrite and ferrite. Free lime and alumina were found in the clinker without a dopant, indicating incomplete clinker formation.

The incorporation of CaF_2_, P_2_O_5_ and Na_2_O had a significant effect on the phase compositions of the cement clinkers. In the presence of these dopants, free lime was not observed, indicating that these dopants improved the burnability of the raw mix. The added dopants were able to enter the solid solution on the basis of oxide compositions [25,26,27]. CaF_2_ can be utilized as a mineraliser during the clinkering process. When CaF_2_ was added to the raw mixes, the intensity of ternesite increased with the corresponding decrease of the anhydrite and belite diffraction peak intensity. The ye’elimite peak became stronger with the increase of the CaF_2_ dosages. Similarly, the addition of P_2_O_5_ enhanced the formation of ternesite and ye’elimite (Figure 2b). It was indicated that P_2_O_5_ and F can facilitate the formation of ye’elimite in CSA cements [28]. A comparison between P_2_O_5_ and CaF_2_ suggests that P_2_O_5_ is more beneficial for the promotion of the formation of ternesite. The formation of ternesite and ye’elimite also increased with the addition of Na_2_O (Figure 2c). When the content of Na_2_O was 0.3%, the most effective promotion of the formation of ternesite was achieved.

Fe_2_O_3_ and MgO did not demonstrate a notable influence on the formation of ternesite (Figure 2d,e). The free lime peak was still detected when these two dopants were incorporated into the cement clinkers. The addition of Fe_2_O_3_ promoted the formation of ye’elimite, while the intensity of ye’elimite slightly decreased in the presence of MgO. It was shown that Fe_2_O_3_ could substitute Al_2_O_3_ in $C_{4}A_{3}\bar{S}$ to form C4A3−FxxS¯, and the incorporation of Fe_2_O_3_ promoted the formation of cubic ye’elimite [29].

Table 5 presents the mineralogical compositions of cement clinkers doped with CaF_2,_ P_2_O_5_, Na_2_O, Fe_2_O_3_ and MgO. The ye’elimite content of these doped clinkers was close to the targeted content of TCSA cement clinkers. It could be seen that small amounts of anhydrite were found in clinkers doped with CaF_2,_ P_2_O_5_, and Na_2_O, resulting in a high percentage (~30%) of ternesite. The clinker doped with Na_2_O showed the highest content of ternesite. Free lime was found in the clinkers doped with Fe_2_O_3_ and MgO. Compared with the clinkers doped with CaF_2,_ P_2_O_5_, and Na_2_O, more anhydrite and a much lower content of ternesite formed in these two clinkers. The results indicate that the addition of CaF_2_, P_2_O_5_ and Na_2_O can promote the formation of ternesite at 1150 °C, and can thus achieve the coexistence of ternesite and ye’elimite. Moreover, Na_2_O is the most effective candidate among the five dopants.

#### 3.1.2. Influence of the Gypsum Content in the Raw Mixes on the Clinker Composition

**Figure 3:**
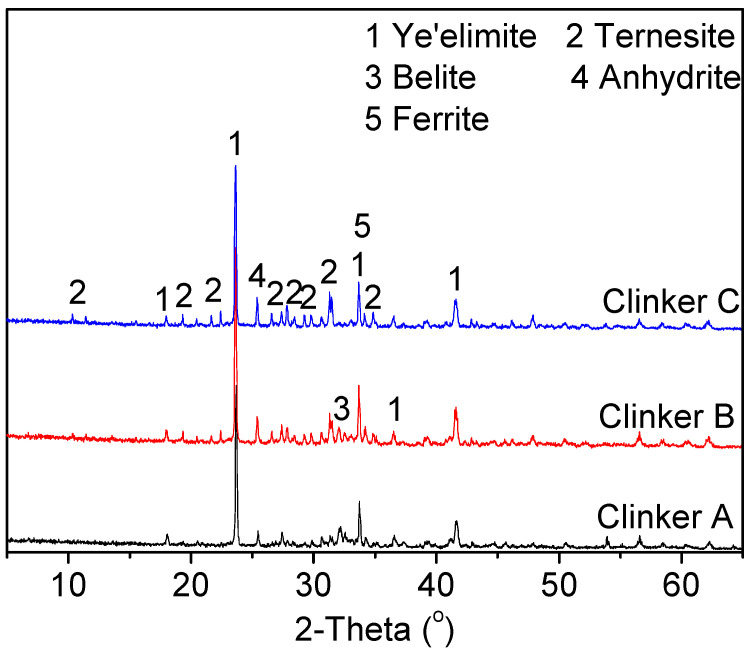
XRD patterns of cement clinkers produced with different amounts of gypsum in the raw mixes.

It was reported that the formation of ternesite is associated with the burning temperature and proportioning of raw materials [7,30]. The SO_3_ to Al_2_O_3_ ratio had a significant effect on the formation of ternesite during the preparation of CSA cement clinkers [17]. Figure 3 shows the XRD patterns of cement clinkers produced with different amounts of gypsum in the raw mixes. These clinkers were doped with 0.3% Na_2_O and sintered at 1200 °C for 30 min. The intensity of the ternesite increased with the increase of the gypsum amounts in the raw mixes. Excessive gypsum content in raw mixes may cause a much lower content of ternesite due to the high quantities of anhydrite present in the cement clinkers (clinker C). Accordingly, the decrease in the belite diffraction peak intensity can be clearly observed. Moreover, increasing the gypsum content in raw mixes promoted the formation of ye’elimite. Considering the complete formation of ternesite and ye’elimite, the gypsum content in the raw mixes should be moderate, like the proportion of clinker B. Therefore, clinker B was further analyzed in the following study.

#### 3.1.3. Influence of the Burning Conditions on the Clinker Composition

**Figure 4:**
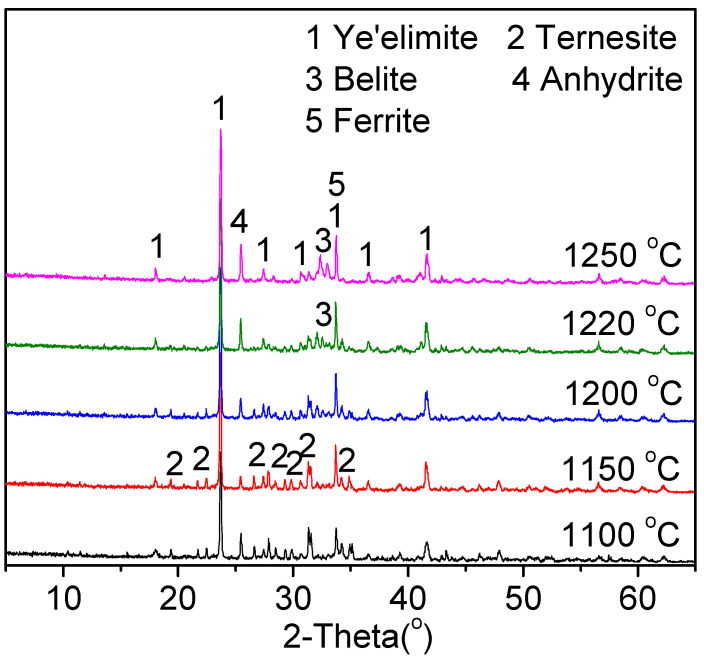
XRD patterns of cement clinker B burned at different temperatures for 30 min.

**Figure 5:**
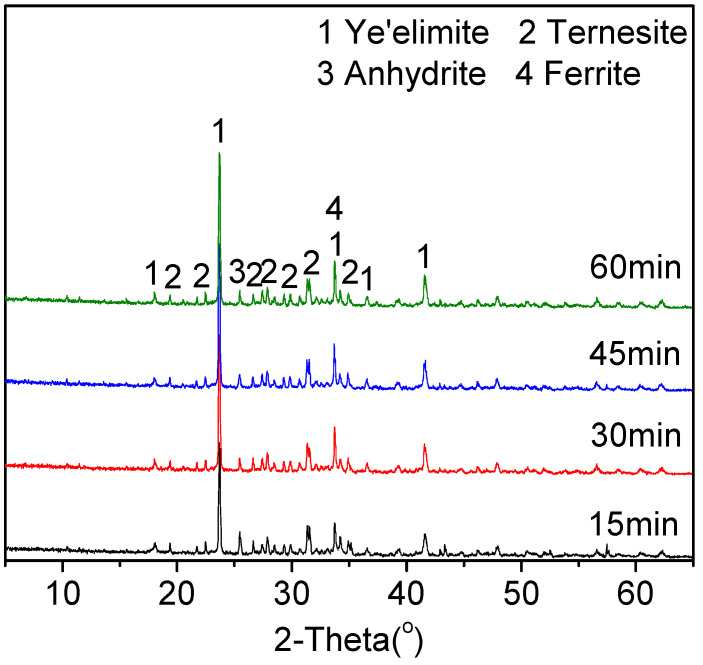
XRD patterns of cement clinker B burned at 1150 °C for different retention times.

The influences of the production parameters, such as the sintering temperature and retention time, were studied in order to produce TCSA cement clinkers. The phase compositions of clinker B with 0.3% Na_2_O obtained under different clinkering conditions are demonstrated in Figure 4 and Figure 5. Table 6 and Table 7 give the quantitative phase compositions of cement clinkers sintered in different conditions. All of the clinkers showed similar phase compositions, ye’elimite, ternesite, ferrite, belite and anhydrite. Free lime was not identified by XRD in the clinkers. In general, adequate proportioning and calcination can be identified by a low free lime content [31]. As shown in Figure 4 and Table 6, when the sintering temperature rose, the intensity of the ternesite increased, while that of anhydrite decreased. The peak of belite was not clearly observed at 1150 °C. As the temperature was higher than 1200 °C, the intensity of ternesite decreased, while that of belite and anhydrite increased. When the temperature rose to 1220 °C, the ternesite peak was not obvious. The peak of ternesite disappeared completely, and the belite peak was significantly strong at the temperature of 1250 °C. Ternesite was stablilised at temperatures between 900 and 1200 °C [32,33,34]. It has been also reported that ternesite could decompose above 1210 °C in the belite-ye’elimite-ternesite clinker [35]. In this experiment, the clinker sintered at 1150 °C showed the highest content of ternesite. Meanwhile, an obvious enhancement in the formation of ye’elimite could be detected with the rise of sintering temperature. When the sintering temperature rose above 1200 °C, the intensity of the ye’elimite was almost constant. Therefore, the clinkers burned at 1150 °C are beneficial to the preparation of the TCSA clinker.

Figure 5 and Table 7 depict the effect of the retention time on the phase compositions of cement clinkers obtained at 1150 °C. There seemed to be little difference in the ternesite peak intensity with the retention time being prolonged. The formation of ternesite was not affected by prolonging the heating time [35]. It was shown that increasing the retention time can promote the formation of ye’elimite. When the retention time was prolonged to 30 min, the intensity of the ye’elimite was almost constant, and simultaneously the anhydrite peak was weakened. Therefore, the suitable retention time appears to be 30 min.

**Figure 6:**
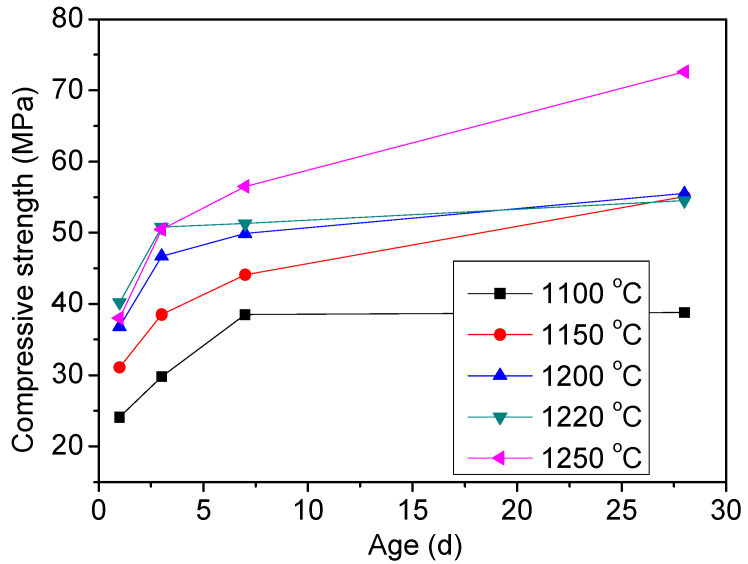
Compressive strength of cement clinker B sintered at different temperatures.

**Figure 7:**
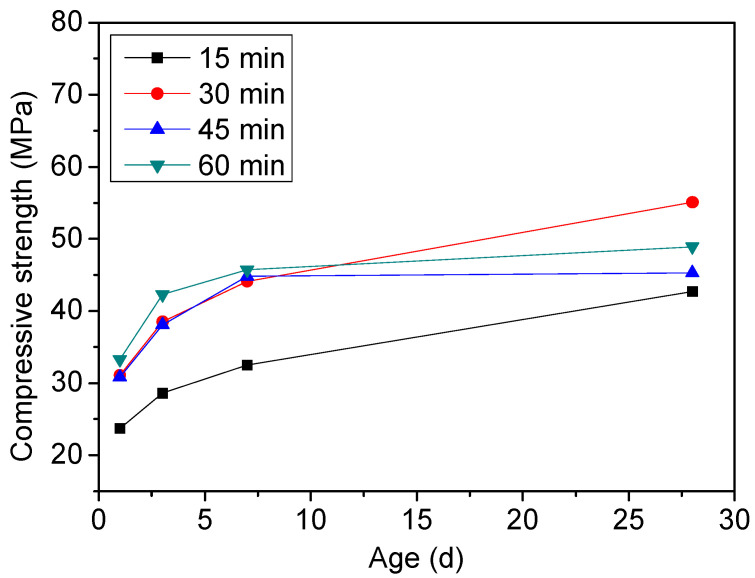
Compressive strength of cement clinker B sintered at 1150 °C for different retention times.

The compressive strengths of clinker B with 0.3% Na_2_O obtained under different clinkering conditions are presented in Figure 6 and Figure 7. The strength development was enhanced by raising the sintering temperature. The early-age strength development of CSA cement is mainly associated with the hydration of ye’elimite with anhydrite and the precipitation of ettringite [36,37]. For TCSA cement clinkers, the early strength may also be ascribed to the hydration of ye’elimite with anhydrite. The increase of the ye’elimite content caused the improvement of the early strength with the rise of the sintering temperature. When the temperature was higher than 1200 °C, the effect of the sintering temperature on the early-age strength was insignificant due to the constant ye’elimite content. The lower early strength of clinkers sintered at temperatures below 1200 °C may be due to the inadequate anhydrite which was consumed by the formation of ternesite in the clinkers. After 28 days of hydration, the clinker obtained at 1250 °C showed the highest strength of 72.6 MPa, while the strengths of the clinkers sintered at 1150 °C, 1200 °C and 1220 °C were similar, at around 55 MPa. The cement clinkers sintered at 1250 °C could be called BCSA cement clinker due to the absence of ternesite. The highest strength of this cement was mainly due to the hydration of ye’elimite with a large amount of anhydrite (~15%). Compared with this cement, the clinker fried at 1150 °C contained the highest content of ternesite, but the lowest content of anhydrite, which resulted in its much lower strength. The similar strength of clinkers sintered at 1150 °C, 1200 °C and 1220 °C indicated that ternesite could improve the strength development at later ages.

The clinkers burned for 15 min had the lowest strength from 1 d to 28 d (Figure 7). This may be explained by the lower content of ye’elimtie. When the retention time was more than 30 min, not much change was observed in the early-age strength. After 28 days of hydration, the clinker burned for 30 min gained the highest strength. Therefore, the optimum retention time is 30 min, considering the strength development.

#### 3.1.4. Characterization by XRD and SEM

**Figure 8:**
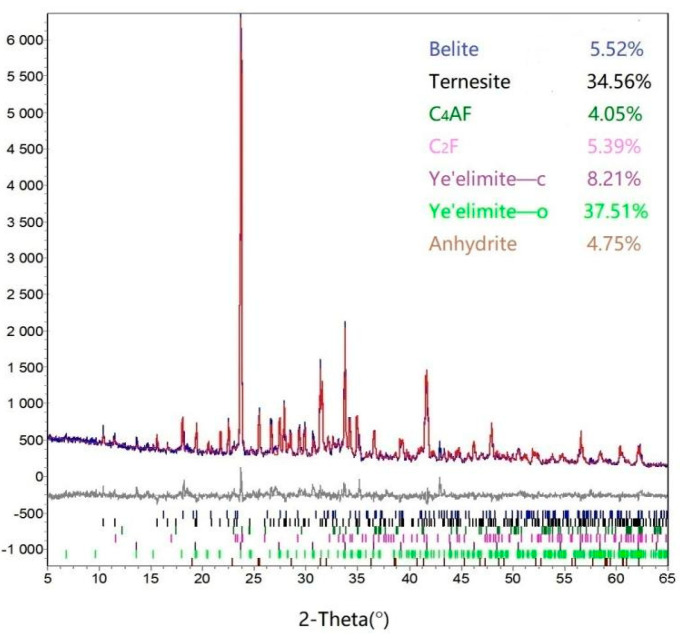
Rietveld refinements for the TCSA cement clinker with 0.3% Na_2_O, R_wp_ = 8.55.

As shown in the above investigation, TCSA cement clinkers can be prepared well at 1150 °C for 30 min by doping with 0.3% Na_2_O. Figure 8 displays the Rietveld refinement plot of the TCSA cement clinker. The main minerals $C_{4}A_{3}\bar{S}$, $C_{5}S_{2}\bar{S}$ and C_4_AF formed in the synthetic TCSA cement clinker. The diffraction peaks of C_2_S and $C\bar{S}$ were also observed, but with low contents. The phase composition of the clinker was somewhat different to the target mineralogical composition. The refinement had a R_wp_ value below 9%, which indicates that the quantitative phase analysis result was highly accurate.

The clinkers obtained at 1250 °C for 30 min were marked as BCSA cement clinkers. Table 8 presents the mineralogical compositions of the TCSA and BCSA cement clinkers. The ye’elimite content of the BCSA cement clinkers was close to that of TCSA cement clinkers. It can be seen that large amounts of belite and anhydrite were formed in the BCSA cement clinkers. The addition of Na_2_O can facilitate the formation of ternesite in TCSA cement clinkers. The ye’elimte content was slightly higher and the anhydrite content was much lower when Na_2_O was added to the TCSA cement clinkers.

**Figure 9:**
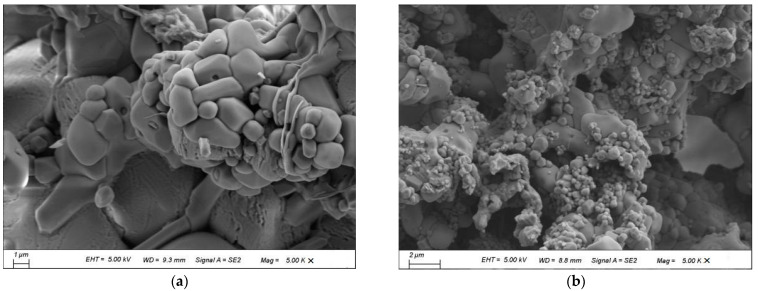
SEM micrographs of cement clinkers with 0.3% Na_2_O. (**a**) BCSA; (**b**) TCSA.

Figure 9 shows the SEM micrographs of cement clinkers which were doped with 0.3% Na_2_O. Rounded particles of belite and polygon ye’elmite could be observed in the BCSA cement clinkers (Figure 9a). Micron-sized rodlike grains of ternesite and rhombic particles of ye’elimite were found in the TCSA cement clinkers (Figure 9b). It is known that the morphology of ye’elimite is a rhombic decahedron [38,39]. The size of the ye’elimite crystals of TCSA cement clinkers seemed to be much smaller than those of BCSA cement clinkers. This may be related to the lower sintering temperature for TCSA cement clinkers.

### 3.2. Performance of the TCSA Cement

#### 3.2.1. Setting Time

The setting time of CSA cements is characterized by the initiation of the solidification and subsequent hardening [40]. Table 9 presents the setting time of TCSA and BCSA cement clinkers. It can be seen that the TCSA cement clinkers exhibited shorter setting times than the BCSA cement clinkers. The hydration reaction of ye’elimite with calcium sulfate is initiated quickly, and promotes the precipitation of ettringite, resulting in the rapid setting of CSA cements [41]. The faster hydration rate of TCSA cement clinkers may be associated with the smaller size of ye’elimite grains. Moreover, the incorporation of ternesite resulted in a great reduction of the setting times of the cements [42]. When Na_2_O was incorporated into the cement clinkers, the setting times of both cement clinkers were significantly shortened. For the TCSA cement clinker, 0.3% Na_2_O decreased the final setting time by 10 min. For the BCSA cement clinker, the same dosage of Na_2_O shortened the initial and final setting times by 20 min. Therefore, a much greater decrease in setting time was achieved for the BCSA clinker than for the TCSA clinker. The ye’elimite content of clinkers doped with Na_2_O was slightly higher than that of clinkers with no dopants. Additionally, the addition of alkali (Na_2_O) can accelerate the hydration of cement [43]. These two aspects may shorten the setting time of cement clinkers.

#### 3.2.2. Compressive Strength

**Figure 10:**
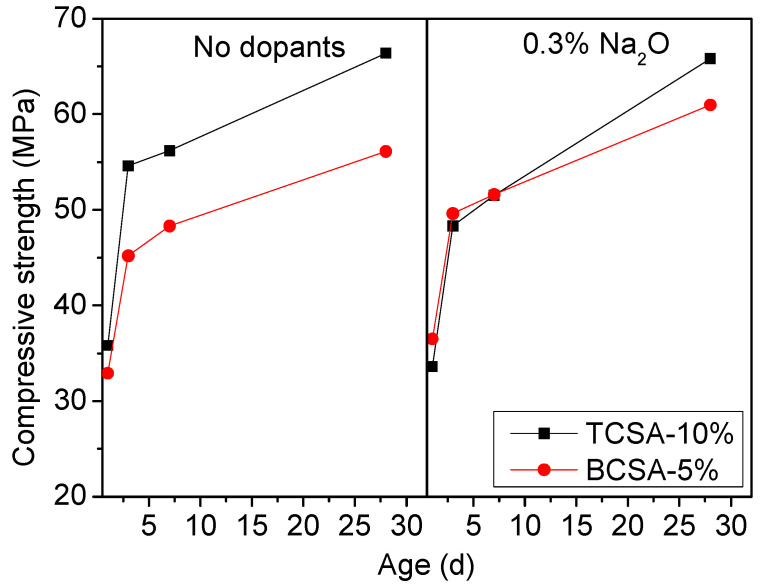
Compressive strengths of TCSA and BCSA cements.

The TCSA and BCSA cement were prepared by mixing the corresponding clinkers with gypsum. Considering that the BCSA cement clinkers contained a large amount of anhydrite which originated from the decomposition of ternesite, the BCSA cements were prepared by mixing the clinkers with 5% gypsum, while the TCSA cements were obtained by adding 10% of gypsum. Figure 10 demonstrates the compressive strength development of the TCSA and BCSA cements. In the absence of dopants, TCSA cement exhibited greater compressive strength than BCSA cement, especially after 3 days of hydration. When Na_2_O was doped during the clinkering, the early strength gain of the TCSA and BCSA cement pastes were similar. The minor difference was that a small decrease in the strength of TCSA cement at 1 d could be found. After 28 days of hydration, the compressive strength of TCSA cement showed a significant increase compared with BCSA cement. It can be concluded that the formation of ternesite can improve the mechanical strength of cement. Previous investigations reported that the incorporation of ternesite could enhance the later strength of BCSA cement [18,44]. The presence of Na_2_O improved the compressive strength of BCSA cement. The addition of alkali (Na_2_O) can accelerate the hydration of cement and raise the degree of reaction [43], which is beneficial to the strength development of BCSA cement. The effect of Na_2_O had different effect on the strength development for TCSA cement. The addition of Na_2_O caused a significant decrease in the strength of TCSA cement at 3 d and 7 d. This may be associated with the phase composition of TCSA clinker. The early strength of TCSA cement may mainly depend on the hydration of ye’elimite with calcium sulfate [21]. The addition of Na_2_O caused a much lower content of anhydrite, which may decrease the early strength of TCSA cement. After 28 days of hydration, the strength gains of TCSA cements with and without Na_2_O were similar. This indicates that a higher content of ternesite increases the later strength of TCSA cement with Na_2_O. It is because of this that the long-term strength of TCSA cement should be studied further.

### 3.3. Hydration of TCSA Cement Pastes

**Figure 11:**
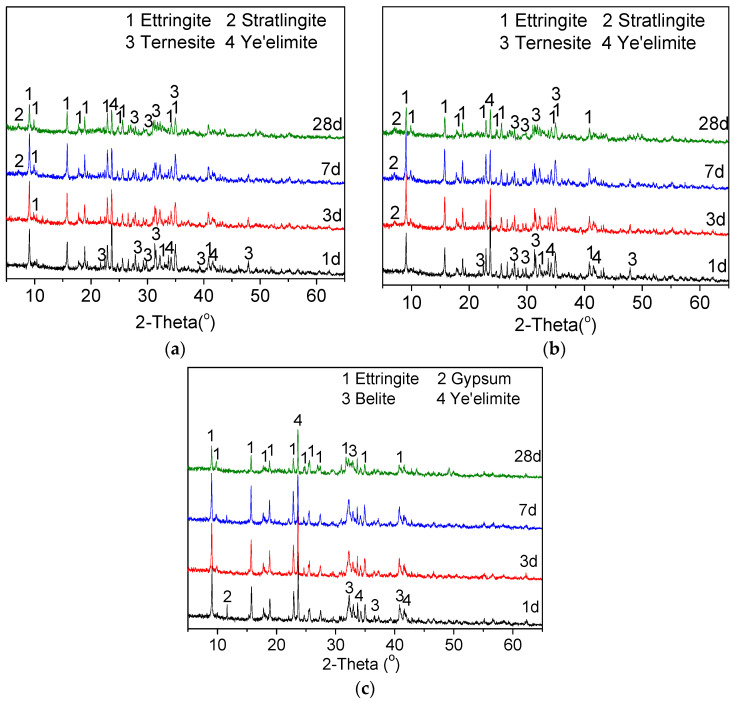
XRD patterns of TCSA and BCSA cement pastes at 1, 3, 7 and 28 days. (**a**) TCSA cement (no dopants); (**b**) TCSA cement (0.3% Na_2_O); (**c**) BCSA cement (no dopants).

The changes of the hydration products were determined using XRD and TG/DSC. Figure 11 gives the XRD patterns of TCSA and BCSA cements hydrated for 1, 3, 7 and 28 days. It can be seen that the TCSA and BCSA cements formed large amounts of ettringite. There were also some unhydrated clinker phases after 28 days of hydration, such as ye’elimite, belite and ternesite. Amorphous hydrated aluminum hydroxide was not found due to its poor crystalline structure [45,46].

For TCSA cement pastes (Figure 11a,b), a significant reduction in the intensity of the ye’elimite peaks was discovered with ongoing hydration. The intensities of the ettringite peaks increased until 7 days of hydration. At 28 days, the decrease of the ettringite peak intensities could be clearly observed. The intensities of the ternesite peaks significantly decreased with time, especially after 28 days of hydration. This suggests that the ternesite formed in TCSA cement clinker is a reactive phase. Traces of strätlingite (C_2_ASH_8_) were found in the TCSA cement pastes. Belite could react with AH_3_ to produce strätlingite in CSA cements after the depletion of calcium sulfate [47]. It was found that the content of strätlingite increased with the decrease of the gypsum content in the CSA cements [48]. The lower content of gypsum in TCSA cement may cause the formation of strätlingite. In addition, the dissolution of ternesite was accompanied by the release of calcium silicate, which would react with AH_3_ to form strätlingite. It can be seen that strätlingite was present in the pastes with 0.3% Na_2_O after 3 days of hydration, while this occurred in the pastes with no dopants at day 7. Compared with the pastes with no dopants, the ettringite peaks were more intense in the pastes with 0.3% Na2O at 3 d and 7 d. The addition of Na_2_O affected the hydration products of TCSA cements, and thereby caused the different strength developments shown in Figure 10. As seen in Figure 11c, BCSA cement displayed a similar ettringite and ye’elimite evolution as for TCSA cement. The ye’elimite peaks were larger than those of TCSA cement with no dopants. Gypsum was observed even after 7 days of hydration, and disappeared at 28 days. No strätlingite was discovered in the BCSA cement pastes.

Table 10 presents the phase compositions (in weight) of TCSA cement doped with Na_2_O, and BCSA cement. The consumption of ye’elimite, anhydrite and gypsum was faster for TCSA cement. The low sintering temperature could improve the hydration rate of ye’elimite [49]. The amount of ternesite decreased between days 1 and 28. This indicates that ternesite participates in the hydration. The release of calcium sulfate from the dissolution of ternesite can promote the formation of ettringite. This resulted in a higher amount of ettringite in the TCSA cement pastes. Moreover, it was found that the reactivity of belite was higher in TCSA cement pastes. This was confirmed by the formation of strätlingite from the reaction of belite with AH_3_. In contrast, the lower reactivity of belite and a complete lack of strätlingite were observed in the BCSA cement pastes.

**Figure 12:**
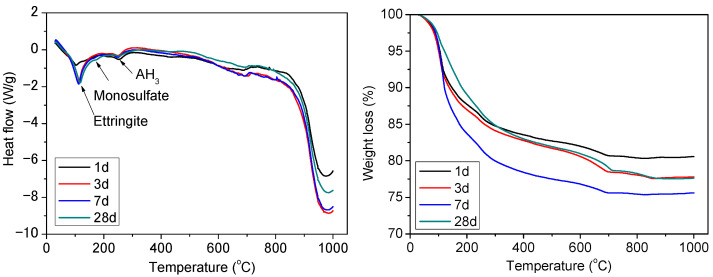
DSC-TG plots of the TCSA cement pastes at 1, 3, 7 and 28 days.

Figure 12 presents the TG-DSC plots of TCSA cements (0.3% Na_2_O) hydrated for 1, 3, 7 and 28 days. All of the pastes exhibited a continuous mass loss at 100–700 °C. The mass loss, centered at ~110 °C, was mainly due to the decomposition of ettringite [50,51]. The second endothermic peak was found between 150 and 220 °C, which was related to the dehydration of strätlingite. Aluminum hydroxide was formed together with ettringite, but it was not detected by XRD, and was identified at approximately 250–300 °C [52,53]. The exothermic peak was observed between 700 and 800 °C, but the phase corresponding with this peak was not clear. Some additional peaks were observed around 900–1000 °C, which may be associated with the presence of carboaluminate hydrate phases. The total mass losses of the cement pastes increased from days 1 to 7, but decreased at 28 days. Gypsum was not detected, in agreement with the XRD findings. This demonstrates that the gypsum from the hydration of ternesite was consumed for the formation of ettringite.

## 4. Conclusions

A new type of ternesite–ye’elimite cement clinker was synthesized by incorporating minor elements. The impact of dopants on the phase formation was investigated in order to attain the coexistence of ternesite and ye’elimite. This paper also aimed to study the cements’ performance. The following conclusions can be made on the basis of this study:

(1) Fe_2_O_3_ and MgO did not demonstrate a remarkable effect on the formation of ternesite. The addition of CaF_2_, P_2_O_5_ and Na_2_O can promote the formation and coexistence of ternesite and ye’elimite at 1150 °C. Na_2_O is the most effective dopant to facilitate the formation of ternesite.
(2) A moderate gypsum content in the raw mixes for clinker B could facilitate the coexistence of ternesite and ye’elimite. A sintering temperature of 1150 °C and a retention time of 30 min were beneficial for the production of TCSA cement clinkers.
(3) The TCSA cement clinkers exhibited shorter setting times than the BCSA cement clinkers. In the absence of dopants, TCSA cement exhibited greater compressive strength than BCSA cement. When Na_2_O was incorporated into the clinkers, the early strength gains of the two cements were similar. After 28 days of hydration, the compressive strength of TCSA cement showed a significant increase compared with BCSA cement.
(4) The dissolution of ternesite could promote the formation of ettringite. The reactivity of belite was higher in TCSA cement due to the formation of strätlingite.

Considering that ternesite will continue to hydrate at later ages, further work will concentrate on the long-term performance of TCSA cements, including its mechanical strength, dimensional stability and ettringite formation.

## Figures and Tables

**Table 1:** Chemical composition of the raw materials determined by XRF (wt.%).

Materials	Limestone	Bauxite	Gypsum	Fly Ash
Oxide				
CaO	44.34	0.38	32.28	4.73
SiO_2_	9.83	12.38	2.02	53.56
Al_2_O_3_	3.25	65.75	0.97	24.01
Fe_2_O_3_	1.83	1.50	0.49	5.91
MgO	3.44	0.30	2.71	0.85
SO_3_	1.06	0.16	39.50	0.51
K_2_O	0.16	1.23	0.12	1.64
Na_2_O	0.13	0.25	-	0.57
TiO_2_	0.28	4.14	0.08	1.38
LOI	35.50	13.45	21.50	4.50

**Table 2:** Theoretical mineralogical compositions of the TCSA clinkers (wt.%).

Phase	A	B	C
$C_{5}S_{2}\bar{S}$	35	48	42
$C_{4}A_{3}\bar{S}$	40	40	40
C_2_S	15	0	0
C_4_AF	10	10	10
$C\bar{S}$	0	2	8

**Table 3:** Proportions of the raw materials (wt.%).

Clinker	Limestone	Bauxite	Fly Ash	Gypsum	Al_2_O_3_
A	59.1	21.5	1.1	18.3	-
B	53.8	20.6	-	24.5	1.1
C	50.3	16.9	-	29.0	3.8

**Table 4:** Weight percentage of the dopant additions (wt.%).

CaF_2_	P_2_O_5_	Fe_2_O_3_	MgO	Na_2_O
0	0	0	0	0
0.5	0.3	0.2	0.2	0.3
1	0.5	0.5	0.5	0.6
	1	1	1	0.9

**Table 5:** Quantitative mineralogical compositions of doped cement clinker A (wt.%).

Phase	0.5% CaF_2_	0.3% Na_2_O	0.3% P_2_O_5_	0.5% Fe_2_O_3_	0.5% MgO
$C_{4}A_{3}\bar{S}$-o	32.2	29.5	30.0	30.5	30.2
$C_{4}A_{3}\bar{S}$-c	6.9	11.3	9.6	10.8	6.7
$C_{5}S_{2}\bar{S}$	25.3	28.5	27.8	16.3	18.5
C_2_S	22.3	18.8	20.7	24.7	26.5
C_4_AF	5.7	6.2	5.9	6.2	5.3
C_2_F	3.9	3.5	3.2	4.3	3.7
$C\bar{S}$	3.7	2.2	2.8	5.6	6.7
f-CaO	-	-	-	1.6	2.1

**Table 6:** Quantitative mineralogical compositions of clinker B sintered at different temperatures (wt.%).

Phase	1100 °C	1150 °C	1200 °C	1220 °C	1250 °C
$C_{4}A_{3}\bar{S}$-o	31.8	37.5	35.4	33.5	32.7
$C_{4}A_{3}\bar{S}$-c	5.3	8.2	7.9	8.8	9.5
$C_{5}S_{2}\bar{S}$	25.7	34.6	29.2	10.3	-
C_2_S	16.4	5.5	10.6	25.7	33.5
C_4_AF	5.6	4.0	3.8	4.3	4.6
C_2_F	4.4	5.4	5.8	4.8	4.3
$C\bar{S}$	9.6	4.8	7.3	12.6	15.4
f-CaO	1.2	-	-	-	-

**Table 7:** Quantitative mineralogical compositions of clinker B sintered at 1150 °C for different retention times (wt.%).

Phase	15 min	30 min	45 min	60 min
$C_{4}A_{3}\bar{S}$-o	31.8	37.5	35.9	35.8
$C_{4}A_{3}\bar{S}$-c	7.1	8.2	8.5	7.8
$C_{5}S_{2}\bar{S}$	32.5	34.6	33.9	34.4
C_2_S	11.7	5.5	6.5	6.7
C_4_AF	3.6	4.0	4.5	4.7
C_2_F	5.8	5.4	5.3	4.9
$C\bar{S}$	7.5	4.8	5.4	5.7

**Table 8:** Quantitative mineralogical compositions of the TCSA and BCSA clinkers (wt.%).

Phase	TCSA (No Dopants)	TCSA (0.3% Na_2_O)	BCSA (No Dopants)	BCSA (0.3% Na_2_O)
$C_{4}A_{3}\bar{S}$-o	36.0	37.5	38.8	39.9
$C_{4}A_{3}\bar{S}$-c	8.4	8.2	6.2	7.5
$C_{5}S_{2}\bar{S}$	30.1	34.6	-	-
C_2_S	8.2	5.5	29.8	28.8
C_4_AF	6.1	4.0	7.2	6.4
C_2_F	3.3	5.4	3.5	3.6
$C\bar{S}$	7.9	4.8	14.5	13.8

**Table 9:** Setting time of TCSA and BCSA cement clinkers.

Clinkers	Initial Setting Time (min)	Final Setting Time (min)
TCSA (no dopants)	20	30
TCSA (0.3% Na_2_O)	17	20
BCSA (no dopants)	55	70
BCSA (0.3% Na_2_O)	35	50

**Table 10:** Quantitative phase compositions of TCSA and BCSA cements (wt.%).

Phase	TCSA (1 d)	TCSA (3 d)	TCSA (28 d)	BCSA (1 d)	BCSA (3 d)	BCSA (28 d)
$C_{4}A_{3}\bar{S}$	7.7	5.5	4.2	11.1	9.8	7.5
$C_{5}S_{2}\bar{S}$	26.2	22.7	19.5	-	-	-
C_2_S	4.6	2.1	-	25.5	25.3	24.6
$C\bar{S}$	0.5	-	-	2.7	1.3	-
$C\bar{S}H_{2}$	1.3	-	-	5.2	3.4	-
Ettringite	33.8	36.3	30.6	30.7	33.6	31.2
strätlingite	-	3.4	6.7	-	-	-
Amorphous	25.4	28.2	30.6	23.9	26.2	30.4

## Data Availability

The data presented in this study are available on request from the corresponding author.
